# Relative Age in School and Suicide among Young Individuals in Japan: A Regression Discontinuity Approach

**DOI:** 10.1371/journal.pone.0135349

**Published:** 2015-08-26

**Authors:** Tetsuya Matsubayashi, Michiko Ueda

**Affiliations:** 1 Osaka School of International Public Policy, Osaka University, Toyonaka, Osaka, Japan; 2 Department of Political Science, Syracuse University, Syracuse, NY, United States of America; Hunter College, UNITED STATES

## Abstract

**Objective:**

Evidence collected in many parts of the world suggests that, compared to older students, students who are relatively younger at school entry tend to have worse academic performance and lower levels of income. This study examined how relative age in a grade affects suicide rates of adolescents and young adults between 15 and 25 years of age using data from Japan.

**Method:**

We examined individual death records in the Vital Statistics of Japan from 1989 to 2010. In contrast to other countries, late entry to primary school is not allowed in Japan. We took advantage of the school entry cutoff date to implement a regression discontinuity (RD) design, assuming that the timing of births around the school entry cutoff date was randomly determined and therefore that individuals who were born just before and after the cutoff date have similar baseline characteristics.

**Results:**

We found that those who were born right before the school cutoff day and thus youngest in their cohort have higher mortality rates by suicide, compared to their peers who were born right after the cutoff date and thus older. We also found that those with relative age disadvantage tend to follow a different career path than those with relative age advantage, which may explain their higher suicide mortality rates.

**Conclusion:**

Relative age effects have broader consequences than was previously supposed. This study suggests that policy intervention that alleviates the relative age effect can be important.

## Introduction

A growing body of literature has examined the relationship between children’s relative age in a grade and their academic performance. Evidence collected in many parts of the world suggests that, compared to older students, students who are relatively younger at school entry tend to perform worse on achievement tests [1–3], are more likely to be held back a grade in elementary school [2], and are less likely to attend college [1]. Some studies reported that such “relative age effects” can have long-term consequences, affecting level of income in adulthood [3, 4], whereas others found no long-term wage effects [5, 6].

This study extends the findings of these past studies and examines how relative age in a grade affects suicide mortality rates of adolescents and young adults between 15 and 25 years of age. We focus on mortality by suicide because it is likely to be associated with the mental health of adolescents and young adults, who can be affected by their experiences at school and academic achievement. It is known that academically struggling students are more likely than peers who succeed in academics to suffer from depression [7–9], one of the major factors associated with suicide. Studies also document a direct link between low academic performance and suicidal behaviors [10–12].

Not only can academic disadvantage be a risk factor for students while at school, it also can have long-term negative consequences, which could elevate a risk for suicide [13–15]. Kawaguchi (2011) found that the difference in the years of education at ages 30–34 between those with a relative age disadvantage and those with an advantage was 0.13 and 0.08 for male and female workers, respectively. He also reported that men who were born between January and March earn 3.9% less than those with a relative age advantage (born in April-June), although he found no difference in wage for women [3].

To our knowledge, almost no previous studies have examined the association between children’s relative ages within a cohort and their mortality rates. The only exception is a Canadian study in the late 1990s that examined suicides by individuals less than 20 years of age in Alberta [16]. They found that individuals who had completed suicide were more likely to have been relatively younger than their classmates when they were at school. However, they could not precisely assign individuals to more than 150 school districts in Alberta, where each local authority can set its school entry cutoff date, because they obtained only the addresses of individuals at the time of death. Moreover, the authors could not rule out the possibility that these individuals had received education outside Alberta, making their calculation of relative age even more imprecise. Thus, more accurate estimation is needed in order to fully understand whether children’s birth dates are related to their mortality rates.

In this study, we use data from Japan to compare suicide mortality rates among young individuals who were born right before and after the school entry cutoff date. We investigate whether those who were born right before the school cutoff day and thus youngest in their cohort have higher mortality rates by suicide, as compared to their peers who were born right after the cutoff date and thus older.

Japan is uniquely suited to studying the relative age effect on suicide for three reasons. First, the school entry cutoff date, April 2nd, does not vary by region, and the cutoff date has not changed since 1947. Second, the length of mandatory schooling does not vary by the date of birth, and only the age at which children start school varies by the date of birth. In Japan, children are expected to stay in school until they finish junior high school after 9 years of education, typically at 15 years of age.

Third, most importantly, the Japanese education system allows us to implement a Regression Discontinuity (RD) approach to examine the causal effect of relative age on suicide. In contrast to other countries, late entry to primary school is not allowed in Japan. The School Education Law, enacted in 1947, specifies that the parents of the Japanese citizens shall send their children to elementary school once they turn six years of age. The only exceptions are the cases of severe illness and underdevelopment, but such exclusions are rarely allowed. The fact that parental discretion is not permitted means that selection at school entry is not a concern because the timing of school entry is almost randomly determined by the birth date. This randomization mechanism around the school cutoff date allows us to utilize the RD approach where we isolate the causal effect of relative age on suicide by assuming that individuals who were born right before the school entry cutoff date are unlikely to differ in their baseline characteristics from those who were born immediately after the cutoff date.

## Materials and Methods

### Data

The individual-level mortality data in this study come from the death record in the Vital Statistics of Japan, complied by the Ministry of Welfare, Health and Labour. The Vital Statistics data used in this study were collected by the Ministry of Welfare, Health, and Labour for administrative purposes and were anonymized and de-identified by the Ministry prior to analysis. The use of the Vital Statistics data for this study has been approved by the Ministry of Welfare, Health, and Labour. Informed consent was not obtained from the individuals in the data because the individual records were anonymized before the data were released for public use.

The data in the Vital Statistics are based on death certificates issued by physicians that are subsequently reported to the local government by a family member of the deceased. The records cover all reported deaths in Japan. The information in the death records includes the date of birth, the date of death, age at time of death, occupation groups (in years that end in 0 and 5), marital status, and the underlying cause of death based on the International Classification of Diseases (ICD) 9 standard (until 1994) and the ICD-10 standard (1995-present). We limit our sample to Japanese citizens because it is possible that the non-Japanese went to school under a different education system outside of Japan. Additionally, we focus on death records from 1989 to 2010 for the reason described below.

### The Statistical Model and Variables

Using the individual death records from the Vital Statistics, we compare the mortality rates by suicide among adolescents and young adults who were born just before and after the school entry cutoff date in Japan. More specifically, we estimate the following simple model:

$$Rate_{dob}=\alpha +\beta {\text{After}}_{dob}+\epsilon_{dob},$$ (1)

where *Rate*_*dob*_ denotes the suicide rates of adolescents and young adults whose birth date is *dob*, and *After*_*dob*_ is a dummy variable that takes a value of 1 if *dob* is on or after April 2 in the calendar year and 0 otherwise. We expect that the suicide rates are higher among those who were born just before April 2nd (i.e., right before the cutoff date) than those who were born just after this date. Thus, *β* is expected to be negative.

We choose to use the simple linear model for three reasons. First, the calendar dates, except for the cutoff date, are unlikely to affect the suicide rates. It is difficult to imagine that the suicide rates are linearly or nonlinearly related to the calendar date (i.e., for example, those born on April 10th are not more likely to die by suicide than those born on Aprils 20th). In fact, a local regression line, as shown later, detects no nonlinear relationship between the calendar dates and the suicide rates. Second, our estimation relies on a very small window around the cutoff date and thus a complicated relationship between the forcing variable and the suicide rates is unlikely to exist. Third, using AIC, we confirmed that the linear model is the best fit.

The suicide rates on each date of birth are calculated in the following way:

$$Rate_{dob}=\frac{\sum \;death_{\left[dob,t\right]}}{\sum \;birth_{\left[dob,t\right]}}*100,$$ (2)

where [*dob, t*] denotes the date of birth in year *t*. Because our main focus is on the comparison of the suicide rates by the date of birth of the deceased, we sum up the number of suicides by young individuals with a given date of birth, regardless of their birth year. This creates 365 observations from January 1 to December 31 for each external cause of death. We exclude data on February 29 in leap years from our study. We then divided the total number of suicides for each *dob* by the total number of births on the same calendar day multiplied by 100, creating the rate of suicides for each date of birth. The data on the number of births were also obtained from the Vital Statistics of Japan. Our analysis includes deaths coded as E950-E959 under the ICD9 standard (1989–1994) and X60-X84 under the ICD10 standard (1995–2010).

Note that *death*_*dob*_ is not the number of suicides on a particular day; rather, it is the number of suicides by young individuals whose birthday is *dob*. We calculate *Rate*_*dob*_ for adolescents and young adults who died between 15 and 25 years of age because the relative age effect is most likely to appear around the end of compulsory education (at age 15 in Japan) or while they are still in school and to be less influential after young adults leave school and enter a labor market. It is also because the deaths by intentional injuries are rare for individuals aged less than 15 years. During our study period, the number of suicides among those below 15 years old is about 70 per year.

We analyze individuals who were born during the period of 1974 to 1985 and died at ages 15–25 between 1989–2010. This is because individual birth records are available from 1974 and the latest death records available at the time of this study were from 2010. Table 1 presents the descriptive statistics of the mortality rates by suicide.

**Table 1:** Descriptive Statistics on the Rate of Mortality by Suicide.

	Mean	SD	Min	Max
Suicide	0.126	0.016	0.081	0.190

The sample includes individuals aged between 15 and 25 at the time of death that occurred between 1989 and 2010. The rate of suicide is calculated as the number of suicides divided by the number of total births for each date of birth, multiplied by 100. The number of observations is 365. Source: Birth records (1974–1985) and death records (1989–2010), the Vital Statistics of Japan.

Our empirical strategy rests on the assumption that the timing of births around the school entry cutoff date was randomly determined and therefore that individuals who were born just before and after the cutoff date have similar baseline characteristics. This is a reasonable assumption because, as mentioned previously, parental discretion is minimized by the strict enforcement of the school entry rule. Thus, we take advantage of the school entry cutoff date to implement a RD design. To ensure that our assumption is valid, we fit the above liner regression model within small windows of data centered at the school entry cutoff date. In the discussion section, we consider the possibility that the timing of birth was manipulated around the school entry cutoff date by parents who were aware of the relative age effects.

## Results

**Fig 1:**
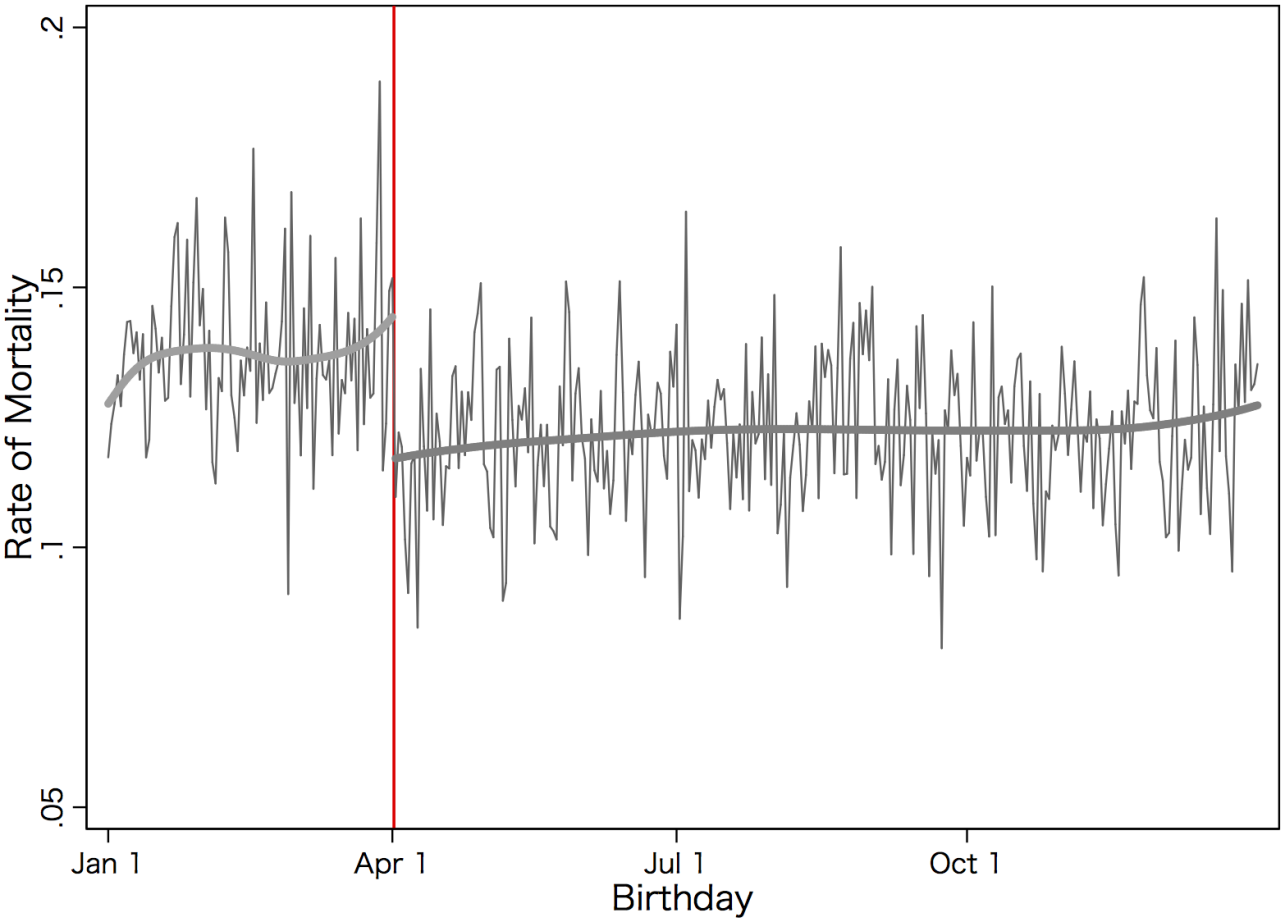
The Rate of Suicide by the Date of Birth. The rate of suicide is plotted against the date of birth. The red line denotes the school entry cutoff date (i.e., Aprils 2nd) in Japan. The gray thick line represents a locally weighted regression line fitted separately before and after the cutoff date. The data include individuals aged between 15 and 25 at the time of death that occurred between 1989 and 2010. Source: Birth records (1974–1985) and death records (1989–2010), the Vital Statistics of Japan.

We begin by graphically examining the patterns of deaths by the date of birth. Fig 1 presents the rates of mortality by suicide plotted against the date of birth. The thick vertical line denotes the school entry cutoff date in Japan, i.e., April 2nd. The gray thick line represents a locally weighted regression line fitted separately before and after the cutoff date.

Fig 1 reveals a clear, discrete break in the suicide rate of young individuals at the school entry cutoff date; those who were born in late March or on the 1st of April were more likely to have died by suicide compared to those who were born on or right after the 2nd of April.

Next, in order to understand the effects of the school entry cutoff more precisely, we turn to the results of our regression analysis. Table 2 displays the estimated coefficients of *β* in Eq (1). The sizes of bandwidth are 7, 14, 21, and 28 days around April 2nd. The results strongly suggest that adolescents and young individuals who were born immediately before the school entry cutoff date are more likely to have died by suicide, as compared to those who were born on or right after that date. According to Column (1) of Table 3, the mortality rate for individuals who were born between March 26th and April 1st is higher by 0.034 than that for those who were born between April 2nd and April 8th. Given that the baseline mortality rate for suicide is 0.126, this is not a trivial difference. The magnitude of the difference decreases as the size of the window widens, but Column (4) suggests that there is a statistically meaningful difference between the groups of individuals who were the oldest and the youngest in class, even when the size of the bandwidth is 28 days.

**Table 2:** The Estimated Effects of School Entry Cutoff Date on the Rate of Suicide.

	Bandwidth			
	(1)	(2)	(3)	(4)
	±7	±14	±21	±28
Suicide	-0.034***	-0.026***	-0.022***	-0.017***
	(0.010)	(0.007)	(0.005)	(0.004)
N	14	28	42	56

All estimates are based on linear regressions using data within ± 7-day, ± 14-day, ± 21-day, ± 28-day windows centered at the school entry cutoff date (April 2). The outcome variable is the rate of mortality by suicide. The mortality rate is calculated as the number of suicides divided by the number of total births on each date of birth, multiplied by 100. The data include individuals aged between 15 and 25 at the time of death that occurred between 1989 and 2010.

*** p <.01 (two-tailed tests).

Source: Birth records (1974–1985) and death records (1989–2010), the Vital Statistics of Japan.

**Table 3:** The Estimated Effects of School Entry Cutoff Date on the Rate of Suicide by Subgroups.

	Bandwidth			
	(1)	(2)	(3)	(4)
	±7	±14	±21	±28
Male	-0.037**	-0.030***	-0.026***	-0.019***
	(0.012)	(0.009)	(0.007)	(0.007)
Female	-0.033*	-0.023**	-0.019**	-0.015**
	(0.015)	(0.009)	(0.007)	(0.006)
Age ≤ 18	-0.005*	-0.002	-0.002	-0.001
	(0.003)	(0.002)	(0.002)	(0.002)
19 ≤ Age ≤ 21	-0.016***	-0.016***	-0.012***	-0.009***
	(0.004)	(0.003)	(0.002)	(0.002)
Age ≥ 22	-0.013	-0.008	-0.008*	-0.007*
	(0.008)	(0.006)	(0.004)	(0.003)
Born in 1974–1980	-0.030**	-0.028***	-0.025***	-0.015**
	(0.013)	(0.009)	(0.007)	(0.006)
Born in 1981–1985	-0.041**	-0.024**	-0.018**	-0.020**
	(0.016)	(0.011)	(0.009)	(0.008)
N	14	28	42	56

All estimates are based on linear regressions using data within ± 7-day, ± 14-day, ± 21-day, ± 28-day windows centered at the school entry cutoff date (April 2). The outcome variable is the rate of mortality by suicide. The mortality rate is calculated as the number of suicides divided by the number of total births by each subgroup on each date of birth, multiplied by 100. The data include individuals aged between 15 and 25 at the time of death that occurred between 1989 and 2010.

*** p <.01,

** p <.05,

* p <.10 (two-tailed tests).

Source: Birth records (1974–1985) and death records (1989–2010), the Vital Statistics of Japan.

The results presented in Table 2 may mask informative patterns across different demographic groups or birth cohorts. Table 3 shows estimation results by gender, by age groups at the time of death, and by birth cohorts. Both male and female individuals who were relatively old in class are less likely to have died by suicide than the youngest ones in class. In addition, the difference between the oldest and the youngest individuals in class is most pronounced among those who died by suicide between ages 19 and 21. We do not see a strong difference in suicide mortality rates based on the date of birth when individuals were younger than 19 years old or older than 21 years old. Finally, the magnitude of the relative age effect does not vary by cohort.

## Discussion

Using individual data of death by adolescents and young adults in Japan from 1989 to 2010, we estimated the effect of relative age within a grade on suicide mortality rates. We exploited the school entry cutoff date, which is April 2nd in Japan, to implement a regression discontinuity design, assuming that the timing of births around the school entry cutoff date was randomly determined and therefore that individuals who were born just before and after the cutoff date have similar baseline characteristics. Our analysis showed that those who were born right before the school cutoff day and thus youngest in their cohort had higher suicide rates at ages between 15 and 25 years old, as compared to their peers who were born right after the cutoff date and thus relatively older.

We conducted three robustness checks of our main findings. First, we verified the assumption that individuals who were born before and after the school entry cutoff are comparable in their baseline characteristics. Our findings were unaffected by selection at school entry because, as explained above, delayed entry to school is not allowed in Japan except for serious medical conditions. However, the timing of birth, rather than the timing of school entry, might be manipulated by parents, which could result in the correlation between parents’ characteristics and their children’s relative age in class. For example, parents with high socioeconomic status might be more likely to be aware of the relative age effects, and might therefore be more likely to ensure that their children were the oldest in class. In other words, individuals who were born right before the cutoff date tended to have parents with low socioeconomic status, while those who were born right after the cutoff date tended to have parents with high socioeconomic status. Because low socioeconomic status is a known risk factor for suicide [13–15], a part of the observed effects might be explained by the gap in parents’ socioeconomic status. In contrast, if such shifts are observed uniformly across different socioeconomic statuses, our main results are attributable to the relative age effect as a result of the school entry cutoff.

To address the possibility that parents with high socioeconomic status were more likely to manipulate the timing of birth, we examined if parents with certain occupations were more likely to shift the timing of birth using occupation data in the Vital Statistics. We used information on the father’s occupation because the occupation of a child’s father is more likely to be indicative of a family’s socioeconomic status for the birth cohorts in our study. Our analysis, as reported in S1 Table, shows no compelling evidence that only parents with certain occupations systematically shifted the timing of birth to ensure that their children were the oldest in class.

Second, we examined the possibility that the sizes of bandwidth we chose (i.e., 7, 14, 21, and 28 days around April 2nd) drove our main findings. We estimated Eq (1) by varying the sizes of bandwidth from ±2 days to ±91 days around April 2nd. The estimation with the smallest bandwidth of ±2 includes 4 observations from March 31 to Aprils 3rd, while the estimation with the largest bandwidth of ±91 includes 182 observations from January 1st to July 1st. Our analysis, as reported in S1 Fig, shows that our main findings hold regardless of the selection of the sizes of the bandwidths.

Third, we investigated whether alternative cutoffs other than April 2nd in the calendar year have a similar effect on the suicide risk of young individuals. If suicide risks change significantly before and after any other calendar dates that are not related to school entry, the observed effects in this study are likely to be explained by other potential mechanisms. To check the possibility that other calendar dates make a difference in the suicidal risks, we estimated Eq (1) by including a dummy variable as *After* that equals 1 if *dob* is on or after each day of the calendar year and 0 otherwise. In other words, we compared the morality rate by suicide before and after all calendar days from January 8th to December 25. Because we set the bandwidth as 7 days before and after the cutoff, our analysis could not use the calendar days from January 1 to January 7 and from December 26 to December 31 as cutoffs. All cutoff dates other than April 2nd can be viewed as a placebo. Our analysis, as reported in S2 Fig, indicates that April 2nd was the only date that created a major gap in the rate of suicides, confirming that the school entry cutoff changed the suicidal risks of young adults between 15 and 25 years old.

While our analysis highlighted the importance of relative age within a grade as a risk factor of youth suicide, the underlying mechanism remains unclear. As discussed in the introduction, one explanation is that children who were among the youngest in class suffered academically during school years and that such academic disadvantage affected their future career choices.

Our supplementary analyses provide further evidence that those who are relatively young in a grade tend to have a lower educational attainment and a different career path than the relatively older ones in the same grade. We compared educational attainment and employment status of all individuals between ages 16 and 25 using the Population Census of Japan in 2000. We used the data of those who were born in March and April because the decennial census contains the information on people’s birth year and month but not the date. Our analyses, as reported in S2 Table, shows that the percent of high school students at ages 16–18 is greater among those born in April than in March by 0.44 point, and the percent of college students or graduates at ages 19–22 and 23–25 is also greater by about 2 points. Thus those who were born before and after the cutoff seem to pursue a different career path, where the latter looks for higher educational attainment than the former.

We also compared the employment statuses and the types of jobs between those who were born in March and April. Our analysis here was restricted to those who were in the labor market, and thus not in school at the time of data collection. When focusing on those at ages 16–18, the percent of those who were unemployed is larger among those born in March, while the percent of those with a full-time job is larger among those born in April. A similar pattern is present for those at ages 19–22 and 23–25, but the difference is smaller. When comparing the types of industry, those born in March are more likely to work in the primary and secondary industry, while those born in April are more likely to work in the service industry. Our analysis using the 2010 Census shows very similar results, as reported in S3 Table.

The above evidence suggests that those with relative age disadvantage tend to follow a different career path than those with relative age advantage. The former is less likely to attend high school or college, and is more likely to have a blue-collar job. This implies that the relative age disadvantage translates into academic disadvantage, which may ultimately affect their socioeconomic status. Individuals with a low socioeconomic status have higher odds for suicidal behavior and completed suicides.

Our study has two major limitations. First, our analysis examined only individuals who died at ages between 15 and 25 years old because the individual-level birth data before 1974 are not available for researchers. It is possible that we obtain different results when we study older individuals. Second, the observed relationship between relative age and suicide rates may exist only in Japan and possibly other East Asian countries, where academic pressure is generally high. It is an important future research agenda to replicate our findings with data from other countries.

The empirical results of this study suggest that relative age effects have broader consequences than previously assumed. This study showed that the relative age at school entry affects mortality rates by suicide, not just academic performance and economic outcomes as the previous research has demonstrated. This study highlighted the importance of policy intervention that alleviates the relative age effect. Given that education at the early stage of life plays an important role in people’s future well-being, the arbitrary cutoff of school entry will generate a life-time disadvantage by the timing of birth for a non-trivial number of people. In the case of Japan, those who were born in February and March will be strongly affected by the current cutoff of April 2nd. To alleviate the negative consequences of the arbitrary cutoff, the government has a few policy options, such as allowing late entry to primary school or special assistance to those born just before the cutoff at the early stage of primary education.

## Supporting Information

S1 Table Estimates of Discontinuities in the Number of Births in Vital Statistics data by Father’s Occupation, 1980 and 1985. This table shows no compelling evidence that only parents with certain occupations systematically shifted the timing of birth to ensure that their children were the oldest in class.
(PDF)

S2 Table Demographic Characteristics of the Population at Ages 16–25 who was Born in March and April, using the 2000 Census. This table shows that those born in March and thus with relative age disadvantage tend to follow a different career path than those born in April and with relative age advantage.
(PDF)

S3 Table Demographic Characteristics of the Population at Ages 16–25 who was Born in March and April, using the 2010 Census. This table replicates the results reported in S2 Table by using the 2010 Census data.
(PDF)

S1 Fig The Effects of Alternative Bandwidths on the Estimation Results. This figure shows that our main findings hold regardless of the selection of the sizes of the bandwidths.
(PDF)

S2 Fig The Effects of Alternative Cutoffs on the Suicide Rates. This figure shows that April 2nd was the only date that created a major gap in the rate of suicides, confirming that the school entry cutoff changed the suicidal risks of young adults between 15 and 25 years old.
(PDF)
