# Supplementary material for: Relative Age in School and Suicide among Young Individuals in Japan: A Regression Discontinuity Approach
Source: PLoS One. 2015 Aug 26;10(8):e0135349. doi: 10.1371/journal.pone.0135349 (PMC4550458; doi:10.1371/journal.pone.0135349)
Supplement: S1 Table — This table shows no compelling evidence that only parents with certain occupations systematically shifted the timing of birth to ensure that their children were the oldest in class. (PDF) [file pone.0135349.s001.pdf]

S1 Table. Estimates of Discontinuities in the Number of Births in Vital Statistics data by Father's Occupation, 1980 and 1985.

Occupation	±7-day		±14-day		Birth moved (%)	Mean daily No. of births
Professional and Engineering	0.448***	(0.0380)	0.321***	(0.0366)	25	571.8
Management	0.502***	(0.0820)	0.393***	(0.0516)	29	88.5
Clerical	0.0687	(0.0472)	−0.00497	(0.0340)	3	791.0
Sales	0.269***	(0.0413)	0.168***	(0.0333)	14	694.2
Agriculture, Forestry, and Fisheries	0.310***	(0.0445)	0.215***	(0.0392)	17	163.7
Transportation and Communication	0.536***	(0.0462)	0.381***	(0.0408)	31	250.2
Production and Manual Labor	0.309***	(0.0455)	0.193***	(0.0369)	17	1117.6
Security Service	0.498***	(0.0530)	0.321***	(0.0511)	28	90.5
Service	0.315***	(0.0480)	0.226***	(0.0394)	17	174.5
Unemployed	0.420***	(0.0849)	0.186**	(0.0752)	23	31.3

Note: Separate estimates of β from Eq. (3) by occupation are reported with standard errors in parentheses. All estimates are based on linear regressions using data within ±7-day or ±14-day windows centered at the school entry cutoff date (April 2). All models include indicators for each day of the week and an indicator for the Spring Equinox day (March 20 in 1980 and March 21 in 1985). The estimated share of births are based on the estimates that used the ±7-day window and is calculated as $exp(\beta/2) - 1$, following Gans and Leigh (2009). $***$ $p < .01$, $**$ $p < .05$, $*$ $p < .10$ (two-tailed tests). Source: Birth records with occupation data, the Vital Statistics of Japan, 1980 and 1985.
