# Supplementary material for: Relative Age in School and Suicide among Young Individuals in Japan: A Regression Discontinuity Approach
Source: PLoS One. 2015 Aug 26;10(8):e0135349. doi: 10.1371/journal.pone.0135349 (PMC4550458; doi:10.1371/journal.pone.0135349)
Supplement: S2 Table — This table shows that those born in March and thus with relative age disadvantage tend to follow a different career path than those born in April and with relative age advantage. (PDF) [file pone.0135349.s002.pdf]

S2 Table. Demographic Characteristics of the Population at Ages 16-25 who was Born in March and April, using the 2000 Census.

2000	March	April	Total
High School Student (16 ≤Age ≤ 18)			
% No	7.82	7.38	7.60
% Yes	92.18	92.62	92.40
	(364813)	(357711)	(722524)
College Student (19 ≤Age ≤ 22)			
% No	69.47	66.69	68.05
% Yes	30.53	33.31	31.95
	(491754)	(511117)	(1002871)
College Student or Graduate (23 ≤Age ≤ 25)			
% No	73.69	71.37	72.50
% Yes	25.31	28.63	27.50
	(417905)	(437374)	(855279)
Employment Status (16 ≤Age ≤ 18 and in the labor force)			
% Unemployed	19.62	18.07	18.83
% Part-time	43.53	43.37	43.45
% Full-time	32.93	34.81	33.90
% Other	3.91	3.74	3.83
	(22800)	(24036)	(46836)
Employment Status (19 ≤Age ≤ 22 and in the labor force)			
% Unemployed	11.18	10.51	10.84
% Part-time	24.33	25.01	24.67
% Full-time	61.57	61.55	61.56
% Other	2.92	2.94	2.93
	(264855)	(268764)	(533529)
Employment Status (23 ≤Age ≤ 25 and in the labor force)			
% Unemployed	8.68	8.34	8.50
% Part-time	11.79	11.35	11.57
% Full-time	76.02	76.62	76.33
% Other	3.51	3.69	3.60
	(340609)	(355155)	(695764)
Industry (Age > 15 and employed)			
% Agriculture, Forestry, and Fisheries	0.94	0.84	0.89
% Mining, Construction, Production	26.61	26.33	26.69
% Service	72.45	72.84	72.65
	(542066)	(560741)	(1102807)

Note: Values in parentheses indicate the number of observations for each group of the sample. All of the differences in the values between the March and April samples are statistically significant with a chi-square test. The data include all Japanese citizens at ages 16-25 as of October in 2000. “Other” in the employment category includes those who are self-employed, work for family business, or work at home. Source: the 2000 Population Census of Japan.
