# Supplementary material for: Relative Age in School and Suicide among Young Individuals in Japan: A Regression Discontinuity Approach
Source: PLoS One. 2015 Aug 26;10(8):e0135349. doi: 10.1371/journal.pone.0135349 (PMC4550458; doi:10.1371/journal.pone.0135349)
Supplement: S3 Table — This table replicates the results reported in S2 Table by using the 2010 Census data. (PDF) [file pone.0135349.s003.pdf]

S3 Table. Demographic Characteristics of the Population at Ages 16-25 who was Born in March and April, using the 2010 Census.

2010	March	April	Total
High School Student (16 ≤Age ≤ 18)			
% No	6.06	5.84	5.95
% Yes	93.94	94.16	94.05
	(298648)	(292967)	(591615)
College Student (19 ≤Age ≤ 22)			
% No	56.96	54.24	55.59
% Yes	43.04	45.76	44.41
	(383611)	(385973)	(769584)
College Student or Graduate (23 ≤Age ≤ 25)			
% No	67.57	65.52	66.54
% Yes	32.43	34.48	33.46
	(310913)	(311658)	(622571)
Employment Status (16 ≤Age ≤ 18 and in the labor force)			
% Unemployed	18.45	18.47	18.46
% Part-time	74.72	72.19	73.41
% Full-time	5.11	7.63	6.41
% Other	1.72	1.71	1.72
	(15309)	(16409)	(31718)
Employment Status (19 ≤Age ≤ 22 and in the labor force)			
% Unemployed	12.26	11.83	12.05
% Part-time	47.58	48.70	48.14
% Full-time	38.34	37.59	37.96
% Other	1.82	1.87	1.85
	(183418)	(183108)	(366526)
Employment Status (23 ≤Age ≤ 25 and in the labor force)			
% Unemployed	11.33	11.09	11.21
% Part-time	26.44	25.36	25.90
% Full-time	60.06	61.31	60.68
% Other	2.17	2.25	2.21
	(233936)	(233619)	(467555)
Industry (Age > 15 and employed)			
% Agriculture, Forestry, and Fisheries	1.92	1.80	1.86
% Mining, Construction, Production	38.10	37.84	37.95
% Service	59.98	60.37	60.19
	(199459)	(197298)	(396757)

Note: Values in parentheses indicate the number of observations for each group of the sample. All of the differences in the values between the March and April samples are statistically significant with a chi-square test. The data include all Japanese citizens at ages 16-25 as of October in 2010. “Other” in the employment category includes those who are self-employed, work for family business, or work at home. Source: the 2010 Population Census of Japan.
