# Supplementary material for: Relative Age in School and Suicide among Young Individuals in Japan: A Regression Discontinuity Approach
Source: PLoS One. 2015 Aug 26;10(8):e0135349. doi: 10.1371/journal.pone.0135349 (PMC4550458; doi:10.1371/journal.pone.0135349)
Supplement: S1 Fig — This figure shows that our main findings hold regardless of the selection of the sizes of the bandwidths. (PDF) [file pone.0135349.s004.pdf]

S1 Fig. The Effects of Alternative Bandwidths.

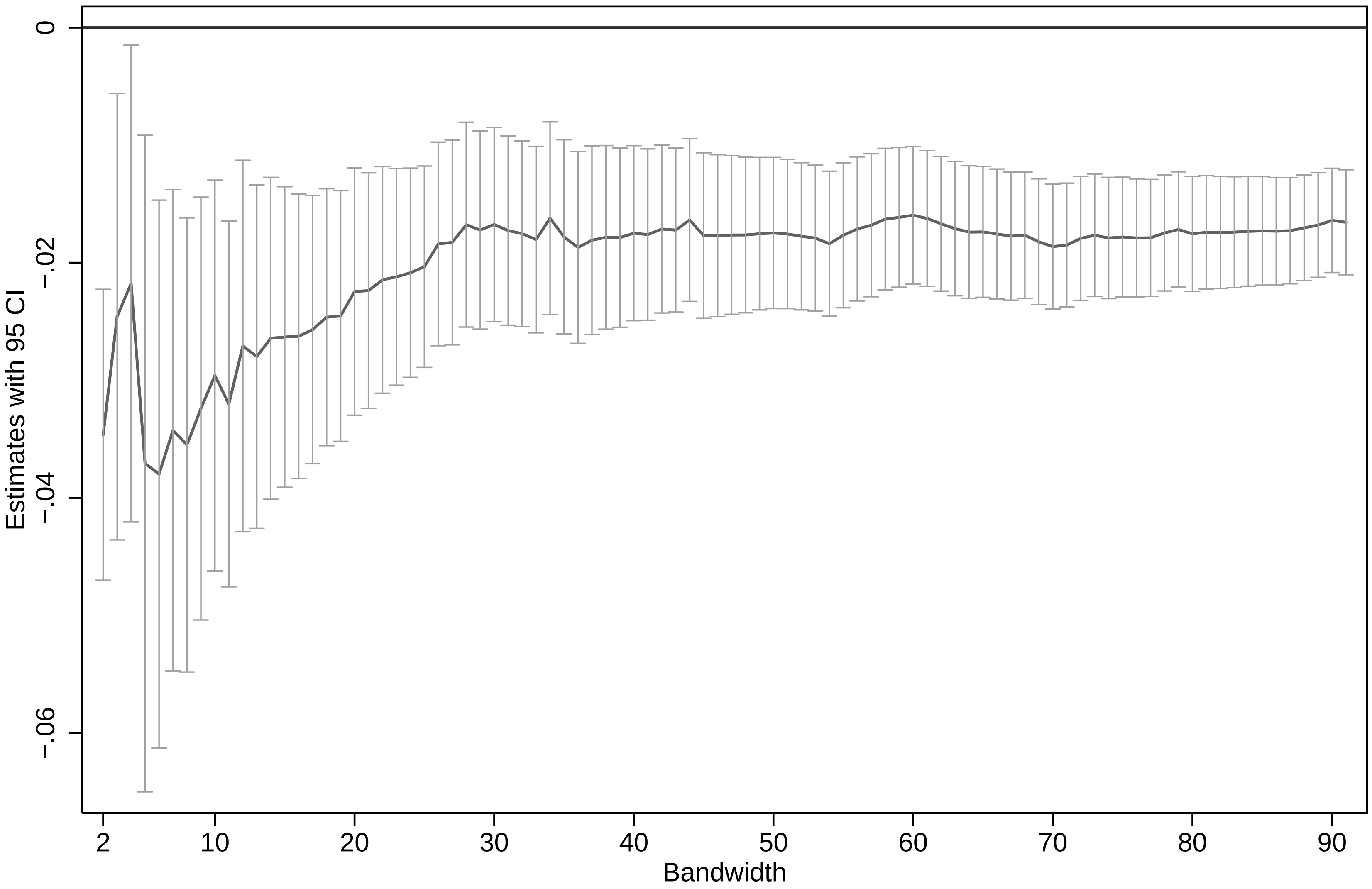

Note: The estimated effects of the school entry cutoff on the rate of mortality by suicide with the 95% confidence intervals are plotted against different bandwidths that range from ±2 to ±91. The estimation with the bandwidth of ±2 includes 4 observations from March 31 to Aprils 3rd, while the estimation with the bandwidth of ±91 includes 182 observations from January 1st to July 1st. Source: Birth records (1974-1985) and death records (1989-2010), the Vital Statistics of Japan.
