# Supplementary material for: Relative Age in School and Suicide among Young Individuals in Japan: A Regression Discontinuity Approach
Source: PLoS One. 2015 Aug 26;10(8):e0135349. doi: 10.1371/journal.pone.0135349 (PMC4550458; doi:10.1371/journal.pone.0135349)
Supplement: S2 Fig — This figure shows that April 2nd was the only date that created a major gap in the rate of suicides, confirming that the school entry cutoff changed the suicidal risks of young adults between 15 and 25 years old. (PDF) [file pone.0135349.s005.pdf]

S2 Fig. The Effects of Alternative Cutoffs on Suicide Rates.

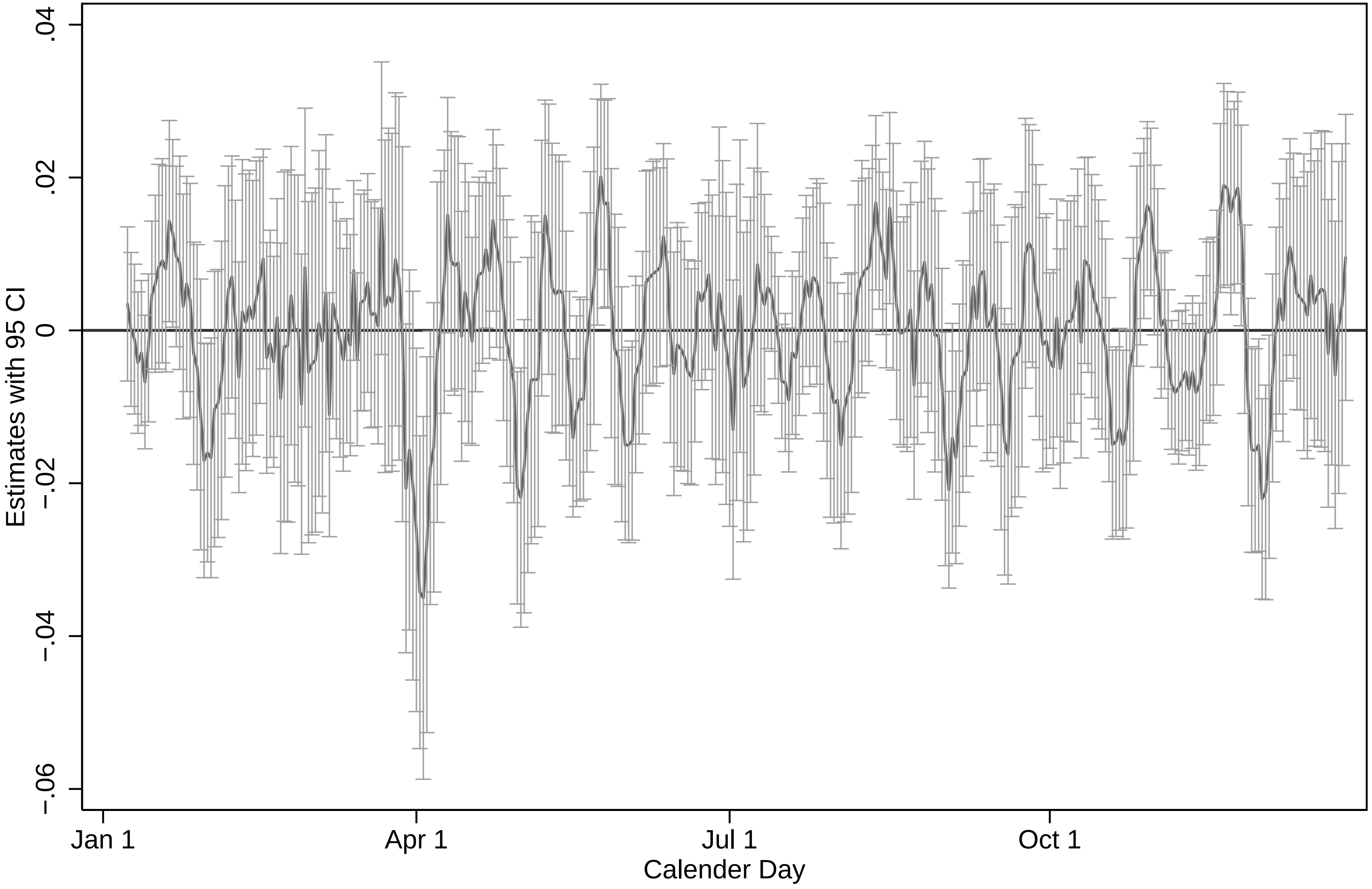

Note: The estimated effects of alternative cutoffs on the rate of mortality by suicide with the 95% confidence intervals are plotted against each calendar day. The bandwidth is set to ±7 days. Source: Birth records (1974-1985) and death records (1989-2010), the Vital Statistics of Japan.
